# Extracellular Vesicles in Cancer Metastasis: Potential as Therapeutic Targets and Materials

**DOI:** 10.3390/ijms21124463

**Published:** 2020-06-23

**Authors:** Akiko Kogure, Yusuke Yoshioka, Takahiro Ochiya

**Affiliations:** Department of Molecular and Cellular Medicine, Institute of Medical Science, Tokyo Medical University, Shinjuku-ku, Tokyo 1600023, Japan; akogure@tokyo-med.ac.jp (A.K.); tochiya@tokyo-med.ac.jp (T.O.)

**Keywords:** extracellular vesicles, cancer metastasis, cancer therapy, cancer biomarker

## Abstract

The vast majority of cancer-related deaths are due to metastasis of the primary tumor that develops years to decades after apparent cures. However, it is difficult to effectively prevent or treat cancer metastasis. Recent studies have shown that communication between cancer cells and surrounding cells enables cancer progression and metastasis. The comprehensive term “extracellular vesicles” (EVs) describes lipid bilayer vesicles that are secreted to outside cells; EVs are well-established mediators of cell-to-cell communication. EVs participate in cancer progression and metastasis by transferring bioactive molecules, such as proteins and RNAs, including microRNAs (miRNAs), between cancer and various cells in local and distant microenvironments. Clinically, EVs functioning as diagnostic biomarkers, therapeutic targets, or even as anticancer drug-delivery vehicles have been emphasized as a result of their unique biological and pathophysiological characteristics. The potential therapeutic effects of EVs in cancer treatment are rapidly emerging and represent a new and important area of research. This review focuses on the therapeutic potential of EVs and discusses their utility for the inhibition of cancer progression, including metastasis.

## 1. Introduction

Metastasis is the primary cause of cancer mortality. It is responsible for 90% of all cancer-related deaths. The metastatic process involves invasion of tumor cells from the primary tumor, intravasation, arrest and extravasation from the circulatory system, followed by angiogenesis and growth at a distant site. Many of the molecular pathways that promote tumorigenesis also promote metastasis and are important in the treatment of both aspects of cancer progression. Although cancer therapies, including chemoradiotherapy, immunotherapy, and molecularly targeted treatment, have been developed, there has been a lack of satisfactory clinical outcomes for patients with cancer metastasis [1,2]. Most therapeutic developments are based on anticancer activity against tumorigenesis and primary growth rather than cancer metastasis. Further clinical therapy applications of agents and preclinical evidence for the effectiveness of these agents against cancer metastasis are still lacking. Therefore, it is important to develop new antimetastatic therapeutics for clinical application.

Cancer metastasis refers to the process by which primary tumor cells invade other sites of the body, proliferate, and finally form new tumors. In the complex tumor microenvironment, cancer cells and surrounding cells (e.g., endothelial cells, fibroblasts, and macrophages) communicate with each other, which creates a metastatic niche and promotes cancer metastasis [3,4]. Recently, “extracellular vesicles” (EVs) have attracted more attention as a novel mode of cell-cell communication. EVs are cell-derived vesicles that contain lipids, proteins, mRNAs, and small RNAs, such as microRNAs (miRNAs). EVs were previously seen as “garbage bags” for the elimination of cellular components. However, in 1996, Raposo et al. showed that EVs derived from B cells induced antigen-specific major histocompatibility complex (MHC) class II-restricted T cell responses [5]. In 2007, Valadi et al. reported that the mRNAs contained in EVs could be translated in recipient cells [6]. Moreover, several groups demonstrated that EV-associated miRNAs were also transferred to recipient cells and led to gene silencing in recipient cells [7,8,9]. These findings suggested that EVs could contribute to the transfer of functional contents, and EVs have rapidly attracted attention as mediators of cell-to-cell communication. Many important biological functions of EVs have been revealed, including functions in cancer, in recent years. Recent work also showed that EVs secreted from various types of cells transport and transfer bioactive molecules, and EVs have attracted broad attention for their ability to regulate metastasis [10,11,12,13]. In this review, we summarize the current knowledge of EVs in cancer metastasis. Furthermore, we discuss the potential use of EVs in clinical treatment of metastasis.

## 2. Extracellular Vesicles (EVs)

### 2.1. Classification and Biogenesis of EVs

EVs are generally classified into at least three types based on their size and biogenesis: exosomes, apoptotic bodies, and microvesicles. Exosomes are approximately 100-nm extracellular vesicles that are generated by the fusion of multivesicular bodies (MVBs) to the plasma membrane [14,15,16,17]. The fusion of the MVB with the plasma membrane is in part regulated by neutral sphingomyelinase 2 (nSMase2), endosomal sorting complex required for transport (ESCRT), syntenin, ALIX, tetraspanins, Rab proteins, and phospholipase D2 [17,18]. Due to their endosomal origin, all exosomes contain membrane transport and fusion proteins (GTPases, Annexins, and flotillin), tetraspanins (CD9, CD63, CD81, and CD82), heat shock proteins (Hsc70, Hsp90, Hsp60m, and Hsp20), proteins involved in multivesicular body biogenesis (Alix and TSG101), and lipid-related proteins and phospholipases [15,17]. Microvesicles are generated by outward budding and fission of the plasma membrane and the subsequent release of these vesicles into the extracellular space. They are usually approximately 1 µm in diameter [19]. Microvesicles are enriched in some lipids and phosphatidylserine [20]. Apoptotic bodies are generally larger than exosomes and microvesicles (µm size) and are produced by dying cells. They contain several intracellular fragments, cellular organelles, membranes, and cytosolic contents [21]. However, it is still difficult to accurately distinguish between different types of vesicles. In this review, we use the term EVs for all types of vesicles present in the extracellular space.

### 2.2. Isolation Methods of EVs

The most popular method of EV isolation remains differential ultracentrifugation (UC) [22]. In the first step, contaminating material is removed through a series of low-speed centrifugation steps. Following this, at higher speeds, the EV fraction is generated. Although UC is a low-cost and simple procedure, making it an ideal means of EV isolation in many labs, nonvesicular proteins are present as contaminants [23]. The combination of UC with other purification methods, such as density gradient centrifugation or ultrafiltration, can increase the purity of the EV fraction [22,24]. Density gradient-based isolation can provide high purification of the EV fraction. However, the method is time-consuming and cannot separate EVs from contaminants of similar density. There are several ways to isolate EVs without using UC. For example, size exclusion chromatography (SEC) is another isolation approach for EVs. The SEC method separates solution molecules based on their size using gel filtration. Several commercial products have been used for EV isolation, such as qEV (IZON) [25]. This method is easy and results in high purity from a small amount of sample, although it is not suitable for a large amount of sample [26]. Because of the presence of many proteins and receptors in the membrane of EVs, immunoaffinity capture can be used to isolate EVs by relying on immunoaffinity interactions between proteins, such as CD63 and CD9, and their antibodies [27]. The combination of immunoaffinity and microfluidic system approaches has been developed for the detection of EV-based diagnostic biomarkers. As immunoaffinity capture can trap specific markers of EVs, the degree of purification could be higher than that of other methods, as mentioned above. However, because the method is unsuitable for EV isolation on a large scale, EVs would need to be preconcentrated with another method. In addition, the interaction between EVs and their antibodies is not easily reversible [22,24,28]. Therefore, the method is limited to downstream experiments. For EVs to be applied in clinical settings, they must be produced at a large scale. Recently, a commercial hollow-fiber bioreactor system has been utilized effectively for large-scale EV production [29,30]. The hollow-fiber bioreactor is based on culturing the cells in hollow and semipermeable fibers, which can attain a higher cell yield in a short incubation time. By scaling up the cell culture, the EV yield could be increased.

Thus, although various isolation techniques have been developed for clinical applications, it is still difficult to obtain both high efficiency and high purity when isolating EVs.

## 3. Function of EVs in Cancer Metastasis

**Figure 1:**
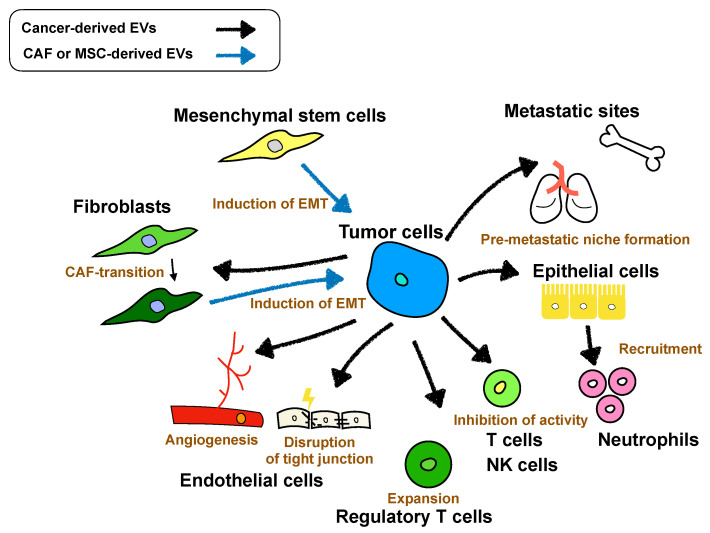
Cell-to-cell communication between tumor cells and surrounding cells via “extracellular vesicles” (EVs) in metastasis. Cancer-derived EVs contribute to cancer metastasis by educating primary and distant tumor environmental cells. In the metastatic process, cancer-derived EVs induce angiogenesis or disrupt tight junctions in ECs, induce cancer-associated fibroblast (CAF) transition, suppress the host immune system, and create a premetastatic niche. Moreover, EVs-derived from cancer surrounding cells, such as CAFs and mesenchymal stem cells (MSCs), also contribute to cancer metastasis.

Crosstalk between primary cancer cells and microenvironmental cells is a key step in cancer metastasis, as described in the Introduction section. Recently, several studies revealed that EVs contribute to distinct steps of cancer metastasis, such as promoting angiogenesis, modulating immune systems, and conditioning the premetastatic niche (Figure 1). In this section, we describe cell-to-cell communication between tumor cells and surrounding cells through EVs.

### 3.1. Function of Cancer-Derived EVs in Angiogenesis

Angiogenesis is a dynamic and tightly regulated process involving angiogenic factors, extracellular matrix components, and endothelial cells (ECs) [31]. Recently, the role of cancer-derived EVs in the process of angiogenesis in primary tumors and distant metastatic sites has attracted attention [32]. Maji et al. showed that metastatic breast cancer-derived EVs, which highly expressed Anx II, promoted angiogenesis in a Matrigel plug assay in vivo [33]. Another group showed that EVs derived from head and neck squamous cell carcinoma regulated in vitro and in vivo tumor angiogenesis through ephrin-B reverse signaling [34]. Furthermore, Wang et al. demonstrated that yes-associated protein (YAP), which plays an important role in angiogenesis, was transferred to HUVECs via lung adenocarcinoma cell-derived EVs [35]. Tight junctions between endothelial cells in brain capillaries are also critical to the metastatic process of cancer cells [36]. Two groups reported that EVs secreted from metastatic breast cancer cells disrupted the formation of tight junctions [37,38]. Thus, metastatic cancer-derived EVs play a role in the promotion of angiogenesis and the destruction of vascular endothelial barriers, which may lead to the promotion of cancer metastasis.

### 3.2. Effect of Cancer-Derived EVs on Host Immune Systems

During the metastatic cascade, tumor cells are exposed to the immune system, which can recognize them and restrict their growth [1]. Recent studies have shown that cancer-derived EVs mediate immunosuppression through various mechanisms [39]. Several reports have revealed that cancer-derived EVs promote the expansion of regulatory T-cells (Tregs). Yen et al. showed that the expression of transforming growth factor (TGF)-β1 in EVs derived from gastric cancer (GC) patients and the ratio of Tregs were both significantly associated with the pathological stages and lymph node metastasis of GC [40]. Their results suggested that immune surveillance can be modulated in GC patients through the induction of Tregs. Another study reported that primary Lewis lung carcinoma (LLC)-derived EVs activated toll-like receptor 3 (TLR3) in lung epithelial cells. Activated TLR3 in lung epithelial cells recruits neutrophils and forms a premetastatic niche in the lung [41]. Cancer-derived EVs also promote myeloid-derived suppressor cell (MDSC) activity, which assists cancer cell dissemination. Huber et al. showed that melanoma-derived EVs mediated MDSC generation from monocytes [42]. Then, the authors incubated EVs purified from conditioned medium from a patient melanoma cell line with monocytes isolated from the peripheral blood mononuclear cells of the same patient and showed that the monocytes inhibited autologous T cell proliferation. In addition, it is well known that cancer-derived EVs suppress natural killer (NK) cell and T cell activity to enhance immune evasion. EVs derived from highly metastatic pancreatic cancer cells [43] and acute myeloid leukemia (AML) [44] interfere with the antitumor activity of NK cells. Ricklefs et al. revealed that cancer-derived EVs suppress T cell activity [45]. Moreover, EV-mediated T cell suppression is due to PD-L1, which is expressed on the surface of cancer-derived EVs [46,47].

These studies suggest that EVs from cancer cells mediate the activation of the immune system to promote cancer metastasis.

### 3.3. Crosstalk between Cancer Cells and Fibroblasts through EVs

Cancer cells educate fibroblasts through EVs, which leads to the progression of metastasis [48]. Cancer-derived EVs are involved in the reprogramming of normal stromal fibroblasts into activated cancer-associated fibroblasts (CAFs) in chronic lymphocytic leukemia [49], hepatocellular carcinoma (HCC) [50], and melanoma [51]. These studies revealed that the CAF transition leads to metastatic niche formation and promotes cancer metastasis. Moreover, several reports have shown that EVs derived from highly metastatic cancer cells are more likely to convert normal fibroblasts into CAFs compared with those derived from non-metastatic cancer cells [50,52].

EVs derived from CAFs also play an important role in cancer metastasis. Kong et al. showed the roles of CAF-derived EVs in premetastatic niche formation [53]. Moreover, Baroni et al. revealed that miR-9 was important for the CAF transition in EVs derived from breast cancer cells [54]. The authors also showed that EVs derived from miR-9-overexpressing normal fibroblasts were taken up by cancer cells, in which they induced tumor cell aggressiveness by modulating genes involved in cell motility and extracellular matrix (ECM) remodeling. These findings suggested that cancer cells and fibroblasts engage in cross talk with each other via EVs to create a metastatic niche.

### 3.4. Premetastatic Niche Formation by Cancer-Derived EVs

A premetastatic niche is a microenvironment prepared for the colonization of circulating tumor cells in specific organs. It is known that premetastatic niche formation is promoted by angiogenesis, CAF transition of fibroblasts, ECM remodeling, and immunosuppression, as mentioned above. Cancer-derived EVs also influence premetastatic niche formation at distant sites. For example, Jung et al. showed that EVs derived from CD44 variant isoform (CD44v6)-positive pancreatic cancer cells contributed to the formation of a premetastatic niche in lymph nodes and lungs [55]. Similarly, another study showed the effect of cancer-derived EVs on lung premetastatic niche formation. Grange et al. reported that treatment with the intravenous injection of EVs derived from CD105-positive renal cell carcinoma modified the lung microenvironment by enhancing the expression of vascular endothelial growth factor (VEGF) and matrix metalloproteinase 2 (MMP2) in lung endothelial cells and that of MMP9 in whole lung tissue [56]. In addition, Peinado et al. also showed the function of cancer-derived EVs in lung premetastatic formation in mice. Intravenously injected EVs derived from highly metastatic mouse melanoma cells were mainly distributed to the interstitium of the lung and bone marrow [57]. Moreover, they found that EVs enhanced lung endothelial permeability and educated bone marrow-derived cells, leading to the creation of a metastatic niche for lung metastasis. These studies indicate that cancer-derived EVs may contribute to metastatic organotropism. Hoshino et al. demonstrated the involvement of EVs as mediators of organotropism of metastasis. EVs derived from a breast cancer cell subline that colonize the lung and brain could home into the corresponding target organs after EV injection. Moreover, EVs significantly increased the lung metastatic capacity of nonmetastatic breast cancer cells in the lung [58]. They also revealed that distant metastases were associated with integrins on the EV surface. In addition, integrin expression patterns play a crucial role in determining the metastatic site. For example, α6β4 and α6β1 are associated with lung metastasis, while integrin αvβ5 is linked to liver metastasis [58]. Thus, cancer-derived EVs have the potential to generate premetastatic niches and regulate metastatic organotropism.

### 3.5. Mesenchymal Stem Cell (MSC)-Derived EVs Contribute to Cancer Metastasis

EVs derived from cancer stromal cells have also been implicated in different stages of cancer metastasis, especially epithelial-mesenchymal transition (EMT). EMT is a key process involved in cancer cell metastasis, during which epithelial cells acquire mesenchymal characteristics and the enhancement of cell motility and migration. The importance of MSCs present in the cancer stroma in EMT induction has been well studied [59,60,61,62]. These studies demonstrated that treatment with MSC culture medium or MSC coculture promoted EMT in breast or gastric cancer cells. Recently, several studies have shown that MSC-derived EVs contain factors that stimulate EMT induction and promote EMT and cancer metastasis. For example, Lin et al. investigated the effect of EVs derived from adipose tissue-derived MSCs on breast cancer cells and demonstrated that EVs promoted the migration of cancer cells via activation of the Wnt signaling pathway [63]. Similarly, Zhou et al. showed that MSC-derived EVs promoted EMT in breast cancer cells. In this study, human umbilical cord-derived MSCs promoted the invasion and migration of breast cancer cells and enhanced the induction of EMT through the ERK pathway [64]. In addition, Zhao et al. found that the EMT-promoting effect in lung cancer was mediated by EVs secreted from human umbilical cord-derived MSCs [65]. The authors also showed that the knockdown of TGF-β in MSCs inhibited the EMT induction ability of MSC-derived EVs. These reports suggest that MSCs present in the tumor stroma can promote EMT induction through the secretion of EVs.

Collectively, these findings on the function of EVs derived from both cancer cells and stromal cells, such as MSCs, in metastasis provide new insights into the potential clinical application of EVs in the treatment of cancer metastasis.

## 4. Therapeutic Implications of EVs

### 4.1. Inhibition of EV Biogenesis and Secretion

As mentioned above, EVs secreted by cancer cells play a pivotal role in promoting cancer metastasis. Therefore, impairing the biogenesis and secretion of EVs by cancer cells might constitute a potential strategy for cancer therapy.

Rab proteins have been shown to be involved in the production of EVs in both normal cells and cancer cells [14,66,67]. Several Rabs have been reported to be involved in cancer development and progression. Bobrie et al. showed that knockdown of Rab27b reduced the numbers of EVs in the culture medium and inhibited the lung metastasis of 4T1 breast cancer cells in mice [68]. The formation and secretion of EVs may be modulated through several approaches. GW4869, a nSMase2 inhibitor, inhibits EV secretion and decreases the metastatic rate [69,70,71]. Fabbri et al. showed that the intravenous injection of GW4869 significantly decreased the number of lung metastases in tumor-bearing mice [72]. This effect was partially rescued when LLC-derived EVs were injected into GW4869-treated mice [72]. Savina et al. showed that EV secretion is induced by an intracellular increase in calcium (Ca2+) [73]. To decrease the level of tumor-derived EV release, Chalmin et al. used dimethyl amiloride, which blocks H+/Na+ and Na+/Ca2+ channels, in tumor-bearing mice. They showed that dimethyl amiloride decreased the secretion of EVs from cancer cells by blocking the channels, which subsequently delayed the growth of cancer cells [74]. Thus, targeting EV biogenesis and secretion may have potential clinical implications for cancer metastatic therapy. However, to date, the mechanisms underlying the biogenesis of different EV subtypes are extremely difficult to define. To target EV biogenesis and secretion for clinical applications, more studies that allow the discrimination of EV subpopulations are needed.

### 4.2. Inhibition of EV Uptake by Recipient Cells

Another option for cancer treatment is to capture and remove circulating cancer-derived EVs. Nishida-Aoki et al. tried to capture circulating EVs derived from cancer cells by using antibodies against human CD9 and CD63 [75]. The treatment of mice with anti-CD9 or anti-CD63 antibodies stimulated EV removal by macrophages. Although this treatment had no effect on the primary tumor, tumor metastasis was significantly reduced. Thus, the removal of cancer-derived EVs could be a novel strategy for therapy for cancer metastasis. Targeting surface proteins on EVs can also inhibit the distribution of EVs to distant sites, leading to blockade of the creation of a premetastatic niche. As we mentioned before, Hoshino et al. showed a correlation between the specific integrins expressed on the EV surface and metastatic tropism [58]. Therapeutic strategies targeting distinct integrin expression patterns may significantly minimize EV uptake, which could inhibit cancer metastasis.

### 4.3. Immunotherapy

An extensive investigation has been carried out to determine the roles of EVs in immunological reactions after Zitvogel et al. reported that EVs are loaded with tumor antigens [76]. Dendritic cell (DC)-derived EVs are enriched in membrane proteins involved in antigen presentation, including MHC class I and II molecules, MHC class I-like molecule (CD1), and costimulatory molecules (CD80 and CD86), which induce T-cell activation [77,78]. Therefore, their incorporation by cancer cells could turn them into immunogenic targets. In fact, DC-derived EVs have been used in phase I trials in melanoma, colorectal cancer (CRC), and non-small-cell lung cancer (NSCLC) patients [79,80,81,82]. These trials demonstrated the safety of the use of DC-derived EVs in antitumor treatments in patients. Besse et al. showed the capacity of DC-derived EVs to boost NK cell antitumor immunity in patients with advanced NSCLC in a phase II clinical trial [79]. Thus, the activation of antitumor-specific T cell responses by DC-derived EVs has been proposed to play a key role in the suppression of established tumor growth. Recently, immunotherapy has shown effectiveness in patients with metastatic cancers [83]. Therefore, EV-based immunotherapy could also effectively prevent or inhibit cancer metastasis.

### 4.4. Encapsulation

EVs have emerged as delivery vesicles for small RNAs (siRNAs and miRNAs) for therapeutic applications due to their natural role in intercellular RNA transport. In addition, as cancer-derived EVs have a property of organ tropism in relation to metastasis [58], EVs may efficiently reach metastatic sites. Therefore, it is important to develop techniques for the encapsulation of drugs that inhibit the formation of the metastatic niche by EVs and for the delivery of EVs to the metastatic site. Several studies succeeded in encapsulating small molecules, such as siRNAs, and showed that the siRNAs could be delivered to recipient cells. Alvarez-Erviti et al. used EVs from self-derived DCs that express Lamp2, a membrane protein in EVs, fused to the neuron-specific rabies virus glycoprotein (RVG) peptide for siRNA delivery. The intravenously injected EVs delivered glyceraldehyde 3-phosphate dehydrogenase (GAPDH) siRNA specifically to neurons, microglia, and oligodendrocytes in the brain, resulting in specific gene knockdown [84]. They also showed that siRNA against beta-secretase 1 (BACE1) encapsulated in EVs was successfully delivered to neurons and downregulated BACE1 expression [84]. Similarly, Wahlgren et al. tried to use EVs originating from the peripheral blood of healthy donors as carriers of genetic material to target cells. EVs loaded with siRNAs against mitogen-activated protein kinase (MAPK) were able to efficiently knock down the target gene upon their delivery into in vitro monocytes and lymphocytes [85]. In another study, Shtam et al. targeted RAD51 genes with specific siRNAs delivered via EVs derived from HeLa cells and ascitic fluids because they previously revealed that the decrease in the RAD51 protein level induced massive reproductive cell death in most cancer cell types [86]. They showed that EVs effectively delivered siRNAs against RAD51 into target cells, causing selective gene silencing and leading to reproductive cancer cell death by knockdown of RAD51 [87]. These studies used siRNAs encapsulated by the electroporation method. However, electroporation has been reported to show inefficiency in loading EVs with siRNAs due to protein aggregation [88]. Therefore, the optimization of the electroporation method has been performed recently [89,90]. Recently, some research reported that EVs can be used for chemical antitumor drug delivery. Pascucci showed that EVs containing paclitaxel inhibited in vitro tumor cell growth [91]. Another report showed that engineered EVs were injected intravenously into mice and delivered doxorubicin to tumors, resulting in the inhibition of tumor growth [92].

Thus, EVs loaded with small RNAs and anticancer drugs could be a useful material for drug delivery. Research on EVs for clinical use is in the early stage of development. Therefore, much effort needs to be made to guarantee their safe and effective application for therapeutic use. Based on these studies, targeting the metastatic niche using EV-based drug delivery systems could be a new therapeutic approach to combat cancer metastasis.

### 4.5. Biomarkers of Cancer Metastasis

EVs can be easily detected in a variety of body fluids, including blood [93,94], urine [95], and saliva [96,97]. EVs released into body fluids may provide an excellent biomarker for monitoring the emergence, progression, and prognosis of cancer, as well as predicting the efficacy of treatment regimens. In fact, there are many reports regarding the use of EVs as cancer biomarkers [98,99,100,101]. Recently, a correlation between cancer metastasis and cargos in circulating EVs was reported. Several studies have shown the potential of specific EV-associated miRNAs as biomarkers for metastatic cancer patients. Bryant et al. found an association between miR-141 and miR-375 levels in EVs and metastatic prostate cancer [102]. Tokuhisa et al. investigated the miRNA profiles of EVs derived from peritoneal lavage fluid (PLF) samples from GC patients with or without peritoneal dissemination. miR-21 and miR-1225-5p levels in the PLF-derived EVs of GC patients with the highest incidence of peritoneal metastasis were significantly increased compared to those of patients without metastasis [103]. In another study focusing on EVs in PLF, the upregulation of EV-associated miRs (miR-21-5p, miR-92a-3p, miR-223-3p, and miR-342-3p) was observed in GC patients with peritoneal metastasis [104]. miR-21 expression in plasma-derived EVs was also positively correlated with liver metastasis in CRC patients [105]. Tang et al. showed that miR-320d could be a useful biomarker for distinguishing metastatic from nonmetastatic CRC. In addition, the expression levels of miR-320d were not associated with background factors, such as age, sex, drinking status, and histological type [106]. Other small RNAs in EVs also reflect metastatic conditions. Chen et al. compared urothelial carcinoma of the bladder (UCB) tissue and matched nontumor tissue samples by using circular RNA (circRNA) microarrays to identify UCB-specific circRNAs. Then, they identified a novel small RNA, which was termed circPRMT5. Moreover, upregulated expression of circPRMT5 in the tumor tissue was more likely to occur in patients with lymph node metastasis than in patients without metastasis in EVs derived from serum and urine [107]. Liu et al. showed that the increased expression of the EV-associated lncRNA CRNDE-h was significantly correlated with CRC regional lymph node metastasis and distant metastasis [108]. Few studies have reported that proteins on the EV surface also have potential as biomarkers for cancer metastasis. For example, Wang et al. found that, compared with patients with nonmetastatic NSCLC and healthy donors, patients with metastatic NSCLC showed higher expression levels of lipopolysaccharide-binding proteins [109].

Thus, EV-associated RNAs and proteins show potential as biomarkers for cancer metastasis (Table 1). Compared with traditional biomarkers, EVs have some advantages as liquid biomarkers. First, as EVs can be detected from patient body fluids, detection could be noninvasive. Second, EVs are protected from degradation by protein nucleases in the phospholipid bilayer.

Recently, several commercially available tools, such as ExoDx Prostate kit (IntelliScore), have been used as noninvasive risk assessment tools for the detection of high-grade cancer. The kit assays the gene expression of urine EVs by measuring a combination of ETS-related gene (ERG), prostate cancer antigen (PCA)3, and SAM pointed domain containing ETS transcription factor (SPDEF), which detects and stratifies by risk cases of prostate cancer. Moreover, several clinical trials of EV-based biomarkers in cancer diagnosis have been reported [110,111,112] and registered (clinicaltrial.gov, accessed on June 17, 2020). Although the clinical usage of EVs as biomarkers has not yet been fully validated and explored, EVs derived from body fluids could be a good source of biomarkers for the detection of cancer metastasis in the future.

## 5. Conclusions and Perspectives

**Figure 2:**
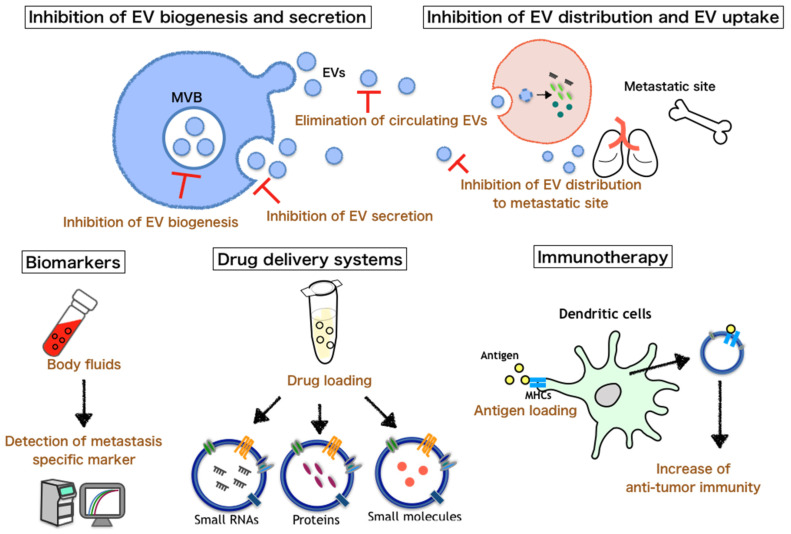
Possible EV engineering for cancer therapy. EVs secreted by cancer cells have a pivotal role in promoting cancer metastasis. Therefore, these EVs could be useful as delivery tools or biomarkers for patients with cancer metastasis.

Over the past few years, many studies have revealed that cancer-derived EVs play a crucial role in cancer metastasis, as mentioned above (Figure 2). In fact, inhibition of EV biogenesis, secretion, distribution, and uptake significantly reduces cancer metastasis in vivo [58,68,69,75]. In addition, the treatment of DC-derived EVs boosts the antitumor immunity of NK cells in cancer patients, though not with metastasis [79]. As immunotherapy is effective in patients with metastatic cancers, DC-derived EV-based immunotherapy is expected to treat cancer metastasis. Moreover, many studies show that EVs can efficiently deliver different kinds of cargo to the target cell. Therefore, EVs are expected to be good delivery materials for therapeutic cargo, such as small RNAs and antitumor drugs, for cancer treatment. However, an efficient EV purification technique and an effective method for loading specific cargos into EVs are required for the use of EVs as delivery tools. In the future, these problems need to be solved.

Moreover, EVs can function as potential biomarkers in the diagnosis and prognosis of cancer metastasis. In particular, EV-associated miRNAs are well documented to reflect the presence of cancer metastasis [98,113,114]. However, for clinical usage, screens of more specific markers and prospective studies are needed. Although, currently, many clinical trials utilizing EV-based technologies in cancer diagnostics are underway compared to those involving cancer therapy, EVs are very promising tools for the diagnosis and also the treatment of cancer metastasis in the future.

## Figures and Tables

**Table 1:** Biomarkers for cancer metastasis in EVs.

Primary Cancer	Metastatic Site	EV Source	Isolation Method	Biomarker	Marker Detection	Reference No.
Prostate cancer	Not mentioned	Plasma	1.2 μm filtration	miR-141 and miR-375	qRT-PCR	[102]
Gastric cancer	Peritoneal dissemination	Peritoneal lavage fluid	UC	miR-21 and miR-1225-5p	qRT-PCR	[103]
Gastric cancer	Peritoneal metastasis	Peritoneal lavage fluid	UC	miR-21-5p, miR-92a-3p, miR-223-3p, and miR-342-3p	qRT-PCR	[104]
Colorectal cancer	Liver metastasis	Plasma	UC	miR-21	tCLN	[105]
Colorectal cancer	Distant metastasis	Serum	UC	miR-320d	qRT-PCR	[106]
Urothelial carcinoma of the bladder	Lymph node metastasis	Serum and urine	UC	circPRMT5	qRT-PCR	[107]
Colorectal cancer	Lymph node metastasis Distant metastasis	Serum	ExoQuick	CRNDE-h	qRT-PCR	[108]

tCLN Tethered Cationic Lipoplex Nanoparticles, UC Ultracentrifugation.

## Abbreviations

EVs	extracellular vesicles
miRNAs	microRNAs
MHC	major histocompatibility complex
MVBs	multivesicular bodies
nSMase2	neutral sphingomyelinase 2
ESCRT	endosomal sorting complex required for transport
UC	ultracentrifugation
SEC	size exclusion chromatography
ECs	endothelial cells
YAP	Yes-associated protein
Tregs	regulatory T-cells
TGF	transforming growth factor
GC	gastric cancer
LLC	Lewis lung carcinoma
TLR	toll-like receptor
MDSC	myeloid-derived suppressor cell
NK	natural killer
AML	acute myeloid leukemia
CAFs	cancer-associated fibroblasts
HCC	hepatocellular carcinoma
ECM	extracellular matrix
VEGF	vascular endothelial growth factor
MMP	matrix metalloproteinase
MSC	mesenchymal stem cell
EMT	epithelial-mesenchymal transition
DC	dendritic cell
CRC	colorectal cancer
NSCLC	non-small-cell lung cancer
RVG	rabies virus glycoprotein
GAPDH	glyceraldehyde 3-phosphate dehydrogenase
BACE1	beta-secretase 1
MAPK	mitogen-activated protein kinase
PLF	peritoneal lavage fluid
UCB	urothelial carcinoma of the bladder
circRNA	circular RNA
ERG	ETS-related gene
PCA	prostate cancer antigen
SPDEF	SAM pointed domain containing ETS transcription factor
